# Effect of Fluoride, Chlorhexidine and Fluoride-chlorhexidine Mouthwashes on Salivary *Streptococcus mutans* Count and the Prevalence of Oral Side Effects

**DOI:** 10.15171/joddd.2015.010

**Published:** 2015-03-04

**Authors:** Fatemeh Sadat Sajadi, Mohammad Moradi, Abbas Pardakhty, Razieh Yazdizadeh, Faezeh Madani

**Affiliations:** ^1^Assistant Professor, Department of Pediatric Dentistry, Faculty of Dentistry & Kerman Oral and Dental Diseases Research Center, Kerman University of Medical Sciences, Kerman, Iran; ^2^Assistant Professor, Department of Medical Microbiology, Medical School & Research Center for Tropical and Infectious Diseases, Kerman University of Medical Sciences, Kerman, Iran; ^3^Professor of Neuropharmacology Institute, Pharmaceutics Research Center, Kerman University of Medical Sciences, Kerman, Iran; ^4^Dentist, Private Practice, Kerman, Iran; ^5^Post-graduate Student, Department of Pediatric Dentistry, Faculty of Dentistry, Kerman University of Medical Sciences, Kerman, Iran

**Keywords:** Chlorhexidine, fluoride, *Streptococcus mutans*

## Abstract

***Background and aims.*** *Streptococcus mutans* is the main pathogenic agent involved in dental caries, and may be eliminated using mouthwashes. The objective of this study was to compare the effects of fluoride, chlorhexidine, and fluoride-chlorhexidine mouthwashes on salivary* S. mutans *count after two weeks of use and determine the prevalence of their side effects on the oral mucosa.

***Materials and methods.*** In this clinical trial, 120 12-14 year-old students were selected and divided into three groups. Each group was given one of fluoride, chlorhexidine, or fluoride-chlorhexidine mouthwashes. They were asked to use it twice a day for two weeks. Salivary samples were collected at baseline and after two weeks. Data were analyzed by Wilcoxon and Kruskal-Wallis tests.

***Results.*** In all the study groups, there were statistically significant reductions in salivary *S. mutans* counts two weeks after using the mouthwashes (P < 0.05). In addition, fluoride-chlorhexidine mouthwash had a significant effect on the reduction of *S. mutans* count in comparison with fluoride alone. The prevalence of oral side effects in fluoride-chlorhexidine mouth-wash was more than 90%.

***Conclusion.*** Adding fluoride to chlorhexidine mouthwash can significantly decrease salivary *S. mutans* count after two weeks. Fluoride-chlorhexidine has the highest rate of oral side effects between the evaluated mouthwash compounds.

## Introduction

Dental caries is a common, chronic and infectious disease. Its etiology is multifactorial and the bacteria have the strongest effect on caries prevalence. *Streptococcus mutans *is the main pathogenic factor in initiating dental caries because it can adhere to tooth surfaces, and produce large amounts of acid.^1,2^ Antibacterial agents can reduce oral bacterial counts and are recommended for prevention of dental caries.^3^ Chlorhexidine is an antibacterial compound against most bacterial species found in the oral cavity.^2^ However, chlorhexidine can cause a change in taste and produce yellow or brown pigments on tooth surfaces. As a result, there is controversy on the use of chlorhexidine for caries prevention.^4^

Fluoride is one of effective agents for caries prevention. Fluoride ions make the tooth structure resistant against demineralization. In addition, they play a role in remineralization of demineralized tooth structure.^2,3,5^ Some studies have shown that fluoride even inhibits colonization, metabolism, and growth of bacteria, preventing plaque maturation and reducing acid production by some species, especially *S. mutans.*^2,3,5^

Each of these compounds have their own mechanisms, but some studies have shown that a combination of these two materials can be effective in prevention of tooth decay.^3^ A study reported that a combination of fluoride and chlorhexidine can have long-term effects in comparison with either of these mouthwashes alone.^6^ Furthermore, the combination of these two compounds has a synergistic effect.^4^Erdem et al^3^ concluded that this combination has a high antibacterial effect.

There is insufficient information regarding the effects of a combination of fluoride-chlorhexidine on salivary *S. mutans.* The aim of this study was to compare the effects of fluoride, chlorhexidine and fluoride-chlorhexidine mouthwashes on salivary *S. mutans* after two weeks and prevalence of their side effects in the oral mucosa.

## Materials and Methods

In this single-blind randomized clinical trial, 120 students, aged 12-14 were selected from a boarding school. Inclusion criteria: proper oral hygiene and a DMFT index of at least 3. Exclusion criteria: receiving any antibiotics or other drugs, chewing gums containing xylitol, fluoride therapy over the past three to four weeks, the existence of any systemic disease, prosthodontic or orthodontic appliances, soft tissue lesions, and rampant caries.^5,7,8^

The study protocol was approved by the Ethics Committee of Kerman University of Medical Sciences (K/90/410). Informed consents were obtained from the parents or legal guardians of the children.

Oral and dental examinations of all the subjects were carried out and the students were trained to brush their teeth twice daily using a toothpaste without fluoride. A period of ten days was considered as “washout” period.^8^ 0.5-1 mL of un-stimulated salivary samples were collected from the subjects; blood agar medium containing crystal violet was used for culturing *S. mutans.* Then, the number of *S. mutans* colony forming units (CFU) per mL of saliva was estimated.

The subject were divided into three groups of 40, and each group was given a type of mouthwash; 10 mL of each mouthwash was used twice daily. The subjects avoided eating and drinking for 30 minutes after mouthwash use.^8^Groups: group A: 0.2% sodium fluoride; group B: 0.2% chlorhexidine gluconate; group C: fluoride-chlorhexidine (0.1% sodium fluoride and 0.2% chlorhexidine). These compounds were made specifically for this study by a pharmacologist.

After two weeks, salivary samples were collected again from the subjects and cultured.^8^

A questionnaire was given to the subjects to assess the impact of the three mouthwashes on taste sensation. The questions were scored according to a scale from −5 (extremely bad) to +5 (extremely good). In addition, the subjects were asked about the side effects of mouthwashes and the questions were scored as follows: short-term anesthesia, long-term anesthesia, blister, mild nausea, none.^5,7-8^

The means and standard deviations of bacterial counts were measured before and after each rinse. Because of non-normal distribution of data, Wilcoxon test was used to compare *S. mutans* counts (CFU/mL of saliva) before and after use of the mouthwashes. In addition, Kruskal-Wallis test was used to compare the three groups. The level of significance for statistical tests was set at P < 0.05.

## Results

Of initial 120 students (mean age, 13 ± 1.4 years), 22, 20, and 36 individuals completed the study course in groups A, B, and C, respectively.

Since the bacterial growth did not have a normal distribution, the median index could better reflect the bacterial growth. Statistical analysis showed that the *S. mutans* count was significantly reduced in all groups (P = 0.001; Table 1).

**Table 1:** Mean and SD of Streptococcus mutans count before and after use of three types of mouthwashes

Groups		Mean (CFU/ml)	Standard Deviation	Median difference	Mean difference
Fluoride	before	6.04×10^5^	1.03×10^6^	1×10^5^	5.95×10^5^
	after	9.09×10^3^	2.30×10^4^		
Chlorhexidine	before	5.15×10^6^	1.01×10^7^	1×10^6^	5.14×10^6^
	after	6.50×10^3^	2.25×10^4^		
Fluoride-Chlorhexidine	before	5.25×10^6^	1.24×10^7^	2×10^6^	5.23×10^6^
	after	2.28×10^4^	5.21×10^4^		
P Value		0.001			

Statistical analysis indicated no significant differences between groups B and C and groups A and B in *S. mutans* reduction (P > 0.05). However, there was a significant difference between groups A and C (P = 0.0001), indicating fluoride-chlorhexidine mouthwash significantly reduced *S. mutans* compared with fluoride mouthwash alone.

Statistical analysis showed no significant difference in the effect on taste sensation between the three mouthwashes (P = 0.084; Table 2). 50% of the subjects reported short-term anesthesia following fluoride-chlorhexidine mouthwash use. 27.30% and 50% reported side effects with fluoride and chlorhexidine mouthwashes, respectively. Only 8.3% of subjects did not report any side effects.

**Table 2:** Frequency of the effect on taste sensation after mouthwash use

Taste sensation	Fluoride		Chlorhexidine		Fluoride-Chlorhexidine	
	number	percent	number	percent	number	percent
Very bad	2	9.1	6	30	15	41.7
Bad	6	27.3	7	35	9	25
Average	8	36.4	5	25	8	22.2
Good	6	27.3	1	5	4	11.1
Very good	0	0	1	5	0	0
Total	22	100	20	100%	36	100

## Discussion

Fluoride, chlorhexidine, and fluoride-chlorhexidine mouthwashes significantly reduced salivary *S. mutans* after two weeks in 12-14-year-old students. Fluoride-chlorhexidine mouthwash significantly reduced bacterial growth compared with fluoride alone. The side effects of fluoride-chlorhexidine mouthwash was significantly more than other mouthwashes.

After chlorhexidine mouthwash use for two weeks,* S. mutans* decreased from 5.15×10^6^ CFU/mL to 6.50×10^3^ CFU/mL which was statistically significant. Chlorhexidine is a cationic composition that can bind to bacterial plaque, hydroxyapatite, and mucous membranes. It can be gradually released and it is effective in decreasing oral bacteria.^9^ Chlorhexidine mouthwash cannot have long-term effects on decreasing oral bacteria, and *S. mutans* increases again after weeks or months.^10^ On the other hand, chlorhexidine has minor anti-caries effect.^11^

In addition, *S. mutans* decreased from 6.04×10^5^CFU/mL to 9.09×10^3^ CFU/mL after using fluoride mouthwash, which was statistically significant.* S. mutans* count has been reported to decrease significantly twenty-four hours after using fluoride varnish.^7^ Similar results have been reported with fluoride mouthwash.^5^Fluoride ions make the tooth structure resistant against degradation by acid and can inhibit bacterial enzymes and change plaque ecosystem. The effect of fluoride ion on carbohydrate metabolism by *S. mutans* has been confirmed.^7^

Furthermore, *S. mutans* decreased from 5.25×10^6^ to 2.27×10^4^ CFU/mL after using fluoride-chlorhexidine mouthwash, which was also statistically significant. This is in line with the results of Erdem et al^3^ They concluded that fluoride-chlorhexidine varnish has more long-term anti-bacterial effects on *S. mutans* than fluoride varnish alone.^3^ Fluoride-chlorhexidine mouthwash has been seen to decrease colonization of *S. mutans* in the dental plaque. In addition, glucose consumption is decreased following fluoride-chlorhexidine mouthwash use.^12^Fluoride-chlorhexidine combination also effectively decreased acid production capability by salivary *S. mutans.*^13^ In addition, some similar studies have confirmed that combination of fluoride-chlorhexidine vanish is more effective on salivary *S. mutans*, caries prevention, phosphorus decrease, potassium metabolism, and acid production by *S. mutans.*^3,6,14^

The results of the present study indicated that 41.7% of individuals in fluoride-chlorhexidine group reported its flavor to be very bad, while 30% and 9.1% of subjects reported the bad taste in chlorhexidine and fluoride groups, respectively. Therefore, most of the participants were dissatisfied with the flavor of fluoride-chlorhexidine mouthwash. In addition, many side effects (in 90% of subjects) were observed using fluoride-chlorhexidine mouthwash. There has been no study on the side effects of fluoride-chlorhexidine mouthwash, but side effects such as tooth discoloration and bad flavor have been reported with chlorhexidine mouthwash.^9^A combination of chlorhexidine and sodium fluoride has been used to decrease side effects of 0.2% chlorhexidine mouthwash.^15^

With regards to effectiveness of fluoride-chlorhexidine mouthwash on salivary *S. mutans*and its anti-caries effects through fluoride, it might be a better compound than the other two mouthwashes evaluated. Compositions such as astringents that include alum, zinc stearate, acetic acid and citric acid may be used to improved its bad flavor.^9^

In this study, we used combinations of fluoride and chlorhexidine mouthwashes which were made in Iran. The tested products demonstrated similar effects compared with known international products, and may be recommended equally where indicated.

Further investigations should be carried out to confirm these results and assess the duration of anti-bacterial and anti-caries effect of fluoride-chlorhexidine mouthwashes.

## Conclusion

Fluoride, chlorhexidine and fluoride-chlorhexidine mouthwashes significantly reduced salivary *S. mutans* counts after two weeks. Fluoride-chlorhexidine mouthwash significantly decreased salivary *S. mutans* counts compared with fluoride alone. The side effects of fluoride-chlorhexidine mouthwash were significantly more than the other two mouthwashes.

## Conclusion

We are thankful to Mr. Adeli for technical assistance in the microbiologic laboratory and Dr. Rad for statistical analysis. Financial support of this study was provided by the Vice Chancellor for Research, Kerman University of Medical Sciences. The authors declare that they have no competing interests.
